# Transcranial Sonography of the Substantia Nigra for the Differential Diagnosis of Parkinson's Disease and Other Movement Disorders: A Meta-Analysis

**DOI:** 10.1155/2021/8891874

**Published:** 2021-04-30

**Authors:** Yan-Liang Mei, Jing Yang, Zheng-Rong Wu, Ying Yang, Yu-Ming Xu

**Affiliations:** ^1^Department of Neurology, The First Affiliated Hospital of Zhengzhou University, Zhengzhou University, Zhengzhou 450000, China; ^2^Department of Transcranial Sonography, The First Affiliated Hospital of Zhengzhou University, Zhengzhou University, Zhengzhou 450000, China

## Abstract

This meta-analysis aimed to evaluate the accuracy of hyperechogenicity of the substantia nigra (SN) for the differential diagnosis of Parkinson's disease (PD) and other movement disorders. We systematically searched the PubMed, EMBASE, Cochrane Library, and China National Knowledge Infrastructure databases for relevant studies published between January 2015 and May 2020. Eligible articles comparing the echogenicity of the SN between patients with PD and those with other movement disorders were screened, and two independent reviewers extracted data according to the inclusion and exclusion criteria. Statistical analyses were conducted using STATA (version 15.0) (Stata Corporation, College Station, TX, USA), Review Manager 5.3 (Cochrane Collaboration), and Meta-DiSc1.4 to assess the pooled diagnostic value of transcranial sonography (TCS) for PD. Nine studies with a total of 1046 participants, including 669 patients with PD, were included in the final meta-analysis. Our meta-analysis demonstrated that hyperechogenicity of the SN had a pooled sensitivity and specificity of 0.85 (0.82, 0.87) and 0.71 (0.66, 0.75), respectively, for distinguishing idiopathic Parkinson's disease from other movement disorders. Furthermore, the area under the curve of the summary receiver operating characteristic was 0.94. Transcranial sonography of the SN is a valuable tool for the differential diagnosis of PD and other movement disorders.

## 1. Introduction

Parkinson's disease (PD) is a common neurodegenerative disease. Its primary motor symptoms include tremor, rigidity, bradykinesia, and postural instability. However, depression, dementia, rapid eye movement sleep disorder, olfactory dysfunction, and other nonmotor symptoms often co-occur in patients with PD [1]. At present, the diagnosis of PD is mainly based on clinical history and motor symptoms. However, several other movement disorders, such as atypical parkinsonism (AP), essential tremor (ET), and secondary parkinsonism, can also manifest with Parkinsonian symptoms, which can complicate the clinical diagnosis of PD [2].

Substantia nigra (SN) hyperechogenicity in patients with PD was first proposed by Becker et al. in 1995 [3]. Although the exact mechanism of SN hyperechogenicity is not clearly understood, it may be related to increased iron content and neuroinflammation in the SN [4]. The transcranial sonographic findings of SN differ between patients with PD and those with other dyskinesia diseases. For example, the main characteristic of PD in transcranial sonography (TCS) is SN hyperechogenicity, while some forms of AP may be characterized by hyperechogenicity of the nucleus lentiformis [5]. Therefore, TCS of the SN may be useful for the differential diagnosis of PD. Previous studies have demonstrated that TCS of the SN can be used to distinguish patients with PD from healthy controls and those with other neurological diseases [6, 7]. However, the sample size of these studies was small, and some studies only explored the diagnostic value of TCS for differentiating between the SN of patients with PD and that of healthy controls. Some previous meta-analyses demonstrated the diagnostic value of TCS for PD [8, 9], but the control groups of the included studies included healthy controls or patients with AP and did not include patients with other types of movement disorders. Therefore, we performed a meta-analysis to assess the value of TCS for the differential diagnosis of PD from other movement disorders.

## 2. Materials and Methods

We conducted this meta-analysis according to the Preferred Reporting Items of Systematic Reviews and Meta-Analyses (PRISMA) statement [10]. The protocol for this systematic review was registered with INPLASY (INPLASY202060068) and is available in full at inplasy.com (https://inplasy.com/inplasy-2020-6-0068/).

### 2.1. Search Strategy

Two independent reviewers (Zheng-Rong Wu and Ying Yang) identified relevant studies published between January 2015 and May 2020 by searching the EMBASE, PubMed, Cochrane Library, and China National Knowledge Infrastructure databases. We searched the existing literature on the diagnostic value of TCS for the differential diagnosis of PD and other movement disorders. Medical Subject Heading terms or keywords including “Parkinson's disease” and “Ultrasonography, Doppler, Transcranial” and entry terms such as “Idiopathic Parkinson's Disease,” “Parkinson's Disease,” “Idiopathic, Parkinson Disease,” “Idiopathic, Primary Parkinsonism,” “Parkinsonism, Primary,” “Transcranial Doppler Sonography,” “Doppler Sonography, Transcranial,” and “Doppler Transcranial Sonography” were used. Moreover, we attempted to acquire unpublished data but were unable to find studies that were appropriate for inclusion.

### 2.2. Selection Criteria

Two reviewers evaluated all the articles independently. Studies were included if they met the following criteria: (1) focused on the diagnostic evaluation of hyperechogenicity of the SN for the diagnosis of PD (only those studies that were published between January 2015 and May 2020 were included); (2) must include participants with PD and other movement disorders; and (3) true positive, false positive, true negative, and false negative cases could be extracted from the studies. Review articles, letters, conference reports, editorial comments, prefaces, and articles not published in English were excluded. The other exclusion criteria were as follows: (1) repeatedly published studies, (2) studies whose full texts were not available, (3) studies whose control groups only contained healthy volunteers, and (4) articles on Parkinsonism but not idiopathic PD.

### 2.3. Data Extraction and Quality Assessment

All the relevant data of the 9 included studies were extracted in a unified manner by two independent reviewers (Zheng-Rong Wu and Ying Yang). Any disagreements were settled by discussion with the third reviewer (Jing Yang). The principal parameters of data extraction included the name of the first author, publication year, number of patients with PD, control group, TCS device, diagnostic criteria for PD, and overall number of true-positives, false-negatives, true-negatives, and false-positives each. The quality of each article was assessed with the revised version of the Quality Assessment of Diagnostic Accuracy Studies (QUADAS-2) tool [11], which enabled assessment of the risk of bias and applicability of the primary investigation. Four domains, including patient selection, index result, reference standard, and flow and timing of the study, were scored in a stepwise manner.

### 2.4. Statistical Analysis

In the present study, data analyses were conducted using the statistical software STATA, version 15.0 (Stata Corporation, College Station, TX, USA), Review Manager 5.3 (Cochrane Collaboration), and Meta-Disc, version 1.4 for Windows (XI Cochrane Colloquium, Barcelona, Spain). We calculated Spearman correlation coefficients between sensitivity and 1−specificity to explore the potential threshold heterogeneity. The Cochrane *Q* statistic and inconsistency index (*I*^2^) of the diagnostic ratio were used to assess the nonthreshold heterogeneity, and the difference was considered to be significant if the *p* value was <0.05 or *I*^2^ >50%. The random effects model (DerSimonian Laird method) was used to calculate the pooled diagnostic accuracy if there was heterogeneity among the studies. Otherwise, the Mantel–Haenszel fixed effects model was utilized. We used sensitivity analysis to investigate nonthreshold heterogeneity. Finally, publication bias was calculated using Egger's test, and *p* values <0.1 were considered to indicate statistical significance.

## 3. Results

### 3.1. Study Selection and Characteristics

**Figure 1:**
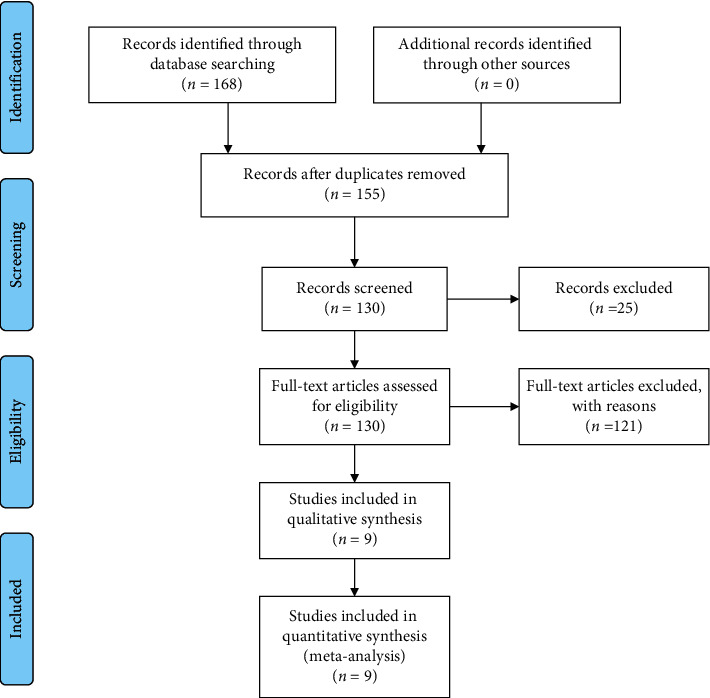
Flow chart of the selection process of the included studies.

A total of 168 related English studies were obtained after a search of the electronic databases. Nine studies were finally selected for inclusion in the meta-analysis based on the inclusion and exclusion criteria. The main study selection process is shown in Figure 1.

### 3.2. Quality Assessment

**Figure 2:**
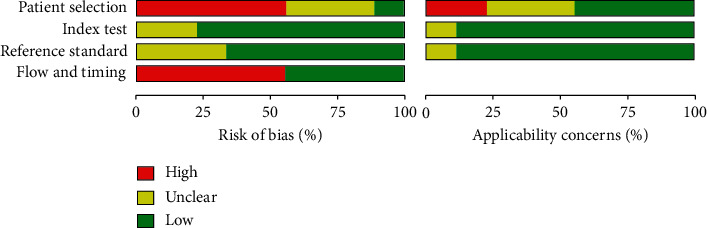
Included studies according to Quality Assessment of Diagnostic Accuracy Studies-2 tool guidelines.

**Figure 3:**
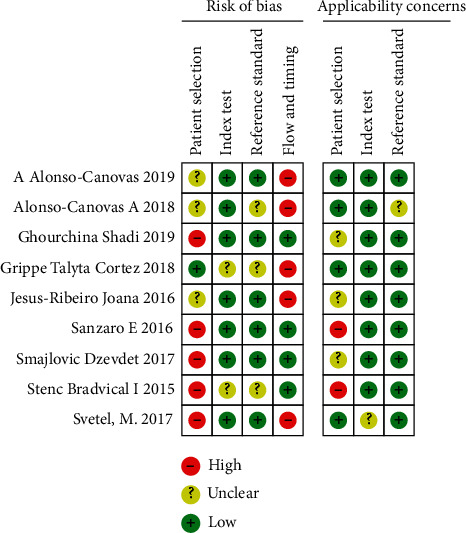
The risk of bias was measured via the Quality Assessment of Diagnostic Accuracy Studies tool.

Study quality assessment was performed using the QUADAS-2 criteria, and the study quality scores ranged from 3 to 5, as shown in Figures 2 and 3. The median quality score was 4 of 7 possible points, and the patient selection domain was the lowest, but none of the eligible articles were excluded because of a poor-quality score.

### 3.3. Basic Characteristics of the Included Studies

Nine related studies [12–20] with a total of 1046 participants, which included 669 patients with PD, published between January 2015 and May 2020, with sample sizes ranging from 35 to 409, were included in the final meta-analysis. The diseases differentiated from PD included atypical Parkinsonism (AP), ET, vascular Parkinsonism (VP), isolated adult-onset focal dystonia (FD), and dopa-responsive dystonia (DRB). The main characteristics of the included studies are summarized in Table 1.

### 3.4. Heterogeneity Analysis

The Spearman correlation coefficient was −0.350 (*p* > 0.05), which indicated that there was no heterogeneity resulting from the threshold effect. However, nonthreshold heterogeneity was detected using the Cochrane Q statistic and the inconsistency index (*I*^2^) of the diagnostic ratio (*I*^2^ = 75.9%, *p* < 0.001).

### 3.5. Diagnostic Accuracy

**Figure 4:**
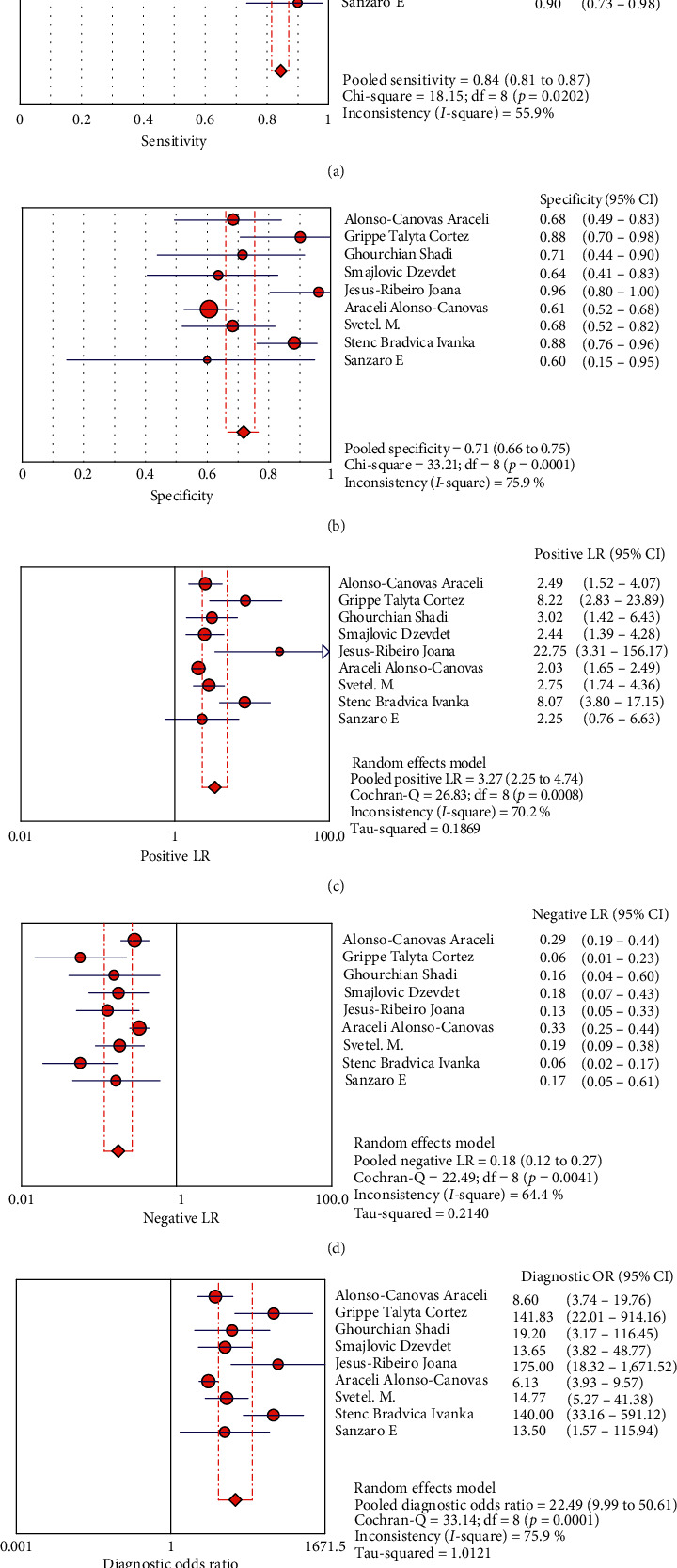
Forest plots of the diagnostic accuracy of transcranial sonography of the substantia nigra in the differential diagnosis of Parkinson's disease.

**Figure 5:**
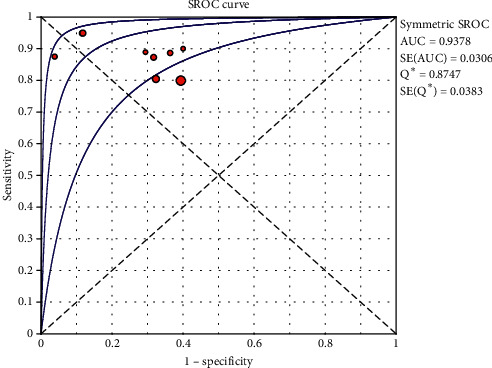
Summary receiver operating characteristic (SROC) curve for transcranial sonography for the differentiation of Parkinson's disease from other movement disorders. AUC = area under curve; SE = standard error; *Q*^*∗*^ = point at which sensitivity and specificity are equal.

The random effects model was used for the statistical analysis because of the heterogeneity resulting from the nonthreshold effect. The pooled sensitivity, specificity, positive likelihood ratio, negative likelihood ratio, diagnostic ratio, and their 95% confidence interval (CI) of TCS for the differential diagnosis of PD and other movement disorders among the 9 studies were 0.85 (0.82, 0.87), 0.71 (0.66, 0.75), 3.27 (2.25, 4.74), 0.18 (0.12, 0.27), and 22.49 (9.99, 50.61), respectively. The forest plots of TCS in the differential diagnosis of PD and other movement disorders are displayed in Figure 4. The area under the curve of the summary receiver operating characteristic (SROC) was 0.94, and the *Q* value was 0.87 (Figure 5).

### 3.6. Subgroup and Sensitivity Analysis

We conducted a subgroup analysis revealing that sample size and TCS device may be the major source of heterogeneity, as shown in Table 2. Sensitivity analysis was conducted by excluding one study at a time and calculating the pooled sensitivity and specificity of the remaining studies. We found that no individual studies significantly changed the pooled sensitivity and specificity, which ranged from 0.83 (0.80, 0.86) to 0.87 (0.84, 0.90) and from 0.68 (0.63, 0.73) to 0.78 (0.72, 0.83), respectively. The sensitivity analysis revealed our meta-analysis had stable and statistically consistent outcomes.

### 3.7. Publication Bias

**Figure 6:**
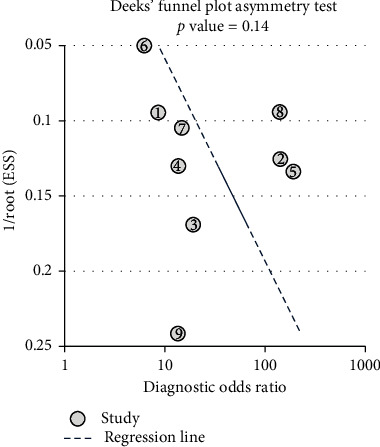
Funnel plot for the assessment of the potential publication bias of the 9 included studies.

The potential presence of publication bias was analyzed using Deeks' funnel plots, which were drawn using STATA 15.0 software (Figure 6). Moreover, we did not detect publication bias in this meta-analysis since the result of Deeks' test was not significant (*p* = 0.14).

## 4. Discussion

The results of our meta-analysis, which included 669 patients with PD from 9 studies, demonstrated a high clinical value of TCS in the diagnosis of PD and other movement disorders. The sensitivity and specificity of TCS ranged from 0.80 to 0.95 and 0.60 to 0.96, respectively, for individual studies. The pooled sensitivity and specificity of TCS for the differential diagnosis of PD were 0.85 and 0.71, respectively. Moreover, the pooled positive likelihood and negative likelihood ratios of this meta-analysis were 3.27 and 0.18, respectively, suggesting that the probability of a positive diagnosis with TCS was 3.27 times higher in patients with PD than that in patients without PD, and the possibility of the correct exclusion of a PD diagnosis was 5.56 times higher than that of a missed diagnosis. Our study demonstrated the utility of TCS in the differential diagnosis of PD. Moreover, the AUC of this meta-analysis was 0.94, which was indicative of a high diagnostic accuracy.

A previously published meta-analysis [8] demonstrated that the pooled sensitivity and specificity of TCS for the differentiation between patients with PD and healthy controls were 0.83 and 0.87, respectively. Their pooled sensitivity was similar to that of our study, while the pooled specificity was higher than that of our study. The main reason is that the control groups in our meta-analysis comprised patients with various movement disorders, such as AP, ET, VP, and DRD, which complicates the final diagnosis of PD.

The pathological change in the SN of patients with PD is the basic principle underlying the diagnosis of PD with TCS [21, 22]. The pathological changes in the SN can also be visualized using magnetic resonance imaging (MRI) [23]. Recent studies have demonstrated degenerative changes in nigrosome-1 in patients with PD, which is characterized by the disappearance of the oval-shaped bright spot in the lateral part of the SN on high-resolution MRI or susceptibility-weighted imaging (SWI) [24–27]. Moreover, they found that this imaging feature could distinguish patients with PD from healthy controls with excellent sensitivity and specificity, which was higher than that of TCS. Xue-Jun Zhao [28] found that 3.0-T SWI can be used to differentiate PD from VP. However, studies by Yun Jung Bae [29] and others [30] have pointed out that MRI cannot effectively differentiate between PD and multiple system atrophy (MSA) and progressive supranuclear palsy (PSP). A previous meta-analysis [31] also indicated that the loss of hyperintensity in the lateral part of the SN could not differentiate PD from AP. However, the primary purpose of our meta-analysis was to confirm the value of TCS in the differential diagnosis of PD from other movement disorders. Our meta-analysis found that TCS had excellent diagnostic accuracy, which can compensate for the limitations of MRI in the differential diagnosis of PD from AP. However, TCS examination is not feasible in all patients because approximately 4–15% of European populations [32, 33] and 15–60% of Asian populations [34] have an insufficient temporal window; thus, MRI of the SN can be used as an adjunct to TCS when necessary.

The principal pathological changes occur in the SN in patients with PD, but TCS can also detect enlargement of the third ventricle in patients of Parkinson's disease dementia (PDD) [35]. In contrast, this phenomenon is seldom found in patients with PD without dementia, indicating that TCS can be used as a potential method for the diagnosis of PDD. TCS can detect a hyperechogenicity of the SN in PD, while the lack of hyperechogenicity in the SN in other movement disorders is the basic principle underlying the ability of TCS to differentiate PD from other movement disorders. Besides, previous studies have shown that the basal ganglia may show hyperechogenicity of TCS in MSA [36]. In comparison, the lack of hyperechogenicity in the basal ganglia of patients with PD indicates that the SN findings combined with basal ganglia echogenicity can better distinguish patients with PD from those with other movement disorders, such as MSA. However, this hypothesis needs to be confirmed by future clinical trials.

The 9 original studies included in this meta-analysis had some heterogeneity, which was mainly caused by nonthreshold effects and may have affected the reliability of the results to some extent. The subgroup analysis results revealed that the sample size and TCS device may be the major source of heterogeneity. We found that the pooled sensitivity and specificity of different age subgroups were similar, indicating the applicability of TCS in the early diagnosis of PD. To our surprise, the results of subgroup analysis showed that the pooled sensitivity and specificity of the subgroup with a smaller sample size (<100) were higher than those of the subgroup with a larger sample size (≥100). We speculated that this may be related to the fact that the control group of the original study with a larger sample size had a greater proportion of other types of Parkinson's syndrome that were not easily differentiated from PD. Sensitivity analysis showed that the pooled sensitivity and specificity did not change dramatically when other studies were excluded one by one.

In conclusion, our meta-analysis suggested that TCS had a high diagnostic accuracy in the differential diagnosis of PD from other movement disorders.

## Figures and Tables

**Table 1:** Characteristics of the included studies.

Author	Year	PD cases	PD age (years)	Control groups	Cutoff value	TCS device	Diagnostic criteria	TP	FP	FN	TN
Alonso-Canovas A	2018	138	71.0 (25–90)	AP, ET, VP	21 mm or 25 mm	2.5 MHz	UK Brain Bank criteria	111	11	27	23
Grippe TC	2018	39	67.0 (17–88)	AP, ET, EPD	20 mm	2.0–3.5 MHz	UK Brain Bank criteria	37	3	2	23
Ghourchian S	2019	18	65.4 (SD 5.8)	PSP	25 mm	2.0–2.5 MHz	UK Brain Bank criteria	16	5	2	12
Smajlović D	2017	44	64.9 (SD 7.8)	PSP, CBD, MSA, VP	20 mm	2.5 MHz	UK Brain Bank criteria	39	8	5	14
Jesus-Ribeiro J	2016	32	62.0 (IQR 13)	ET	24 mm	3.0 MHz	UK Brain Bank criteria	28	1	4	25
Štenc Bradvica I	2015	59	67.2 (SD 7.6)	ET,HCs	20 mm	2.0–4.0 MHz	Not mentioned	56	6	3	45
Alonso-Canovas A	2019	254	69.0 (SD 11)	PSP, CBD, MSA	21 mm or 25 mm	2.5 MHz	UK Brain Bank criteria	203	61	51	94
Svetel M	2017	55	58.9 (SD 10.9)	DRB, FD, HCs	20 mm	2–4 MHz	UK Brain Bank criteria	48	13	7	28
Sanzaro E	2016	30	45.0–85.0	MSA, PSP	25 mm	2.5 MHz	Not mentioned	27	2	3	3

AP: atypical Parkinsonism; ET: essential tremor; VP: vascular Parkinsonism; EPD: excluded PD; PSP: progressive supranuclear palsy; CBD: corticobasal degeneration; MSA: multiple system atrophy; HCs: healthy controls; DRB: dopa-responsive dystonia; FD: isolated adult-onset focal dystonia; TCS: transcranial sonography; TP: true positive; FP: false positive; FN: false negative; PD: Parkinson's disease; TN: true negative; IQR: interquartile range; SD: standard deviation.

**Table 2:** Subgroup analysis results.

Subgroup		*I* ^2^ (%)	Pooled sensitivity	Pooled specificity	Pooled diagnostic odds ratio
Age	≥65.0	84.7	0.83 (0.80, 0.86)	0.70 (0.64, 0.75)	24.14 (7.17, 81.63)
	<65.0	53.4	0.88 (0.81, 0.93)	0.75 (0.65, 0.84)	23.28 (7.01, 77.34)

Sample	≥100	88.1	0.82 (0.78, 0.85)	0.68 (0.61, 0.73)	16.39 (4.07, 66.03)
	<100	0.41	0.89 (0.85, 0.93)	0.77 (0.69, 0.83)	26.96 (11.37, 63.96)

Cutoff value	20 mm	71.1	0.91 (0.87, 0.95)	0.79 (0.71, 0.85)	39.63 (11.17, 140.62)
	Not 20 mm	59.2	0.82 (0.78, 0.85)	0.66 (0.60, 0.72)	12.16 (5.28, 27.98)

TCS device	2.5 MHz	0	0.82 (0.78, 0.85)	0.62 (0.55, 0.69)	7.18 (4.96, 10.39)
	Not 2.5 MHz	62.1	0.91 (0.86, 0.95)	0.83 (0.76, 0.88)	43.35 (22.92, 81.96)

## Data Availability

The data used to support the findings of this meta-analysis are included within the article.
